# Tumor Microenvironment Characterization in Breast Cancer and an Immune Cell Infiltration Score Development, Validation, and Application

**DOI:** 10.3389/fonc.2022.844082

**Published:** 2022-06-27

**Authors:** Lang Li

**Affiliations:** Department of Hematology, Jinhua Hospital of Traditional Chinese Medicine, Jinhua, China

**Keywords:** breast cancer, tumor microenvironment, immune cellular infiltration, tumor mutation burden, immunotherapy, chemotherapy

## Abstract

The tumor microenvironment (TME) refers to the cellular environment in which tumors exist. An increasing number of reports have emphasized its role in tumor progression, prognosis, relapse, metastasis, and therapeutic response with breast cancer (BRCA). Few studies have revealed a systematic landscape of immune cell infiltration (ICI) in BRCA. In this study, we comprehensively analyzed the immune cells infiltrating TME in BRCA. Three ICI patterns were identified through an unsupervised clustering method and an ICI score was developed by a principal component analysis (PCA). A Kaplan-Meier survival with log-rank test revealed a significant overall survival (OS) difference of BRCA patients with these three ICI patterns. We also found that a high ICI score was characterized by an elevated tumor mutation burden (TMB), effector T-cell infiltration, INF-γ-related cytotoxicity, and cytolytic activity score. An independent cohort validated that this ICI score could be a prognostic indicator for BRCA. Two immunotherapeutic cohorts and two chemotherapeutic cohorts confirmed that patients with higher ICI scores showed significant chemotherapeutic and immunotherapeutic advantages. In summary, these results suggest that the ICI patterns could act as a prognostic indicator and that the ICI score could precisely predict the clinical outcome for BRCA patients.

## Introduction

Breast cancer (BRCA) is one of the most common malignancies worldwide with more than 2.2 million newly diagnosed and 680 thousand deaths per year (1). Current therapies have greatly improved the survival rate of early-stage patients. However, the 5-year survival rate remains less than 28% in terminal BRCA patients. To date, the prediction of BRCA prognosis and therapeutic responses mainly depends on the tumour-node-metastasis (TNM) stage. However, BRCA patients in the same TNM stage show different prognoses and therapeutic responses because of tumor heterogeneity. Thus, it is of great importance to incorporate other valuable indicators to predict prognosis and therapeutic responses.

The tumor microenvironment (TME) refers to a cellular environment in which tumors exist, including the surrounding immune cells, stromal cells, and signaling molecules (2–5). Extensive studies have reported a critical role of tumor infiltrating immune components in cancer progression, prognosis, relapse, metastasis, and therapeutic response. For instance, Erdag et al. (6) found that TME differences in immune homing receptors and ligands that influence prognosis in melanoma patients by affecting immune cell recruitment. Kulasinghe et al. (7) reported that tumor-infiltrating lymphocyte-associated biomarkers in the triple-negative breast cancer TME affect chemotherapeutic response and survival. Hu et al. (8) revealed that distinct immune phenotypes have different prognoses, gene mutations, immune infiltrations, and drug sensitivities in TNBC. Risom et al. (9) revealed that myoepithelial disruption in the TME protects BRCA against recurrence *via* multiplexed ion beam imaging by time of flight. Medrek et at. (10) reported that the M2 phenotype tumor-associated macrophages is related to immunosuppressive TME that is permissive to tumor growth and spread. However, one or two single immune components of BRCA are not sufficient to characterize a complex TME. Hence, it is of great significance to investigate the expansive landscape of immune cells infiltrating TME in BRCA.

To date, quantitating the immune cell abundances of tumor tissues in the lab has been a challenge to researchers. A newly issued computational algorithm, single sample gene set enrichment analysis (ssGSEA), can estimate the relative immune cell abundances of bulk RNA-seq samples through a given immune cell gene set (11). This algorithm enables researchers to depict the immune cell infiltration (ICI) landscape. Through this algorithm, previous studies had comprehensively sketched the ICI landscape to predict prognoses and therapeutic benefits in other types of cancers, such as lung adenocarcinoma, gastric cancer, and head and neck squamous cell carcinoma (5, 12, 13). However, a comprehensive ICI landscape in the TME with BRCA has not yet been fully elucidated.

In this study, we compared immune cell abundances and stromal cells contents in TME among tumor with normal tissues. Additionally, we systematically characterized ICI patterns in the TME with tumor tissues using the TCGA-BRCA cohort. Through principal component analysis (PCA), we defined an ICI score to quantify the ICI status for each sample. An independent cohort, METABRIC, confirmed that the ICI score was a robust prognostic tool for BRCA patients. We found that a high ICI score was related to upregulated tumor mutation burden (TMB), effector T-cell infiltration, INF-γ-related cytotoxicity, and cytolytic activity. Two chemotherapeutic cohorts confirmed that patients with high ICI scores had a higher responder rate to chemotherapy (GSE5462 cohort: responder rate [RR], 35.71% in the high ICI group versus 20.83% in the low ICI group; chi-square test, *P* < 0.001; GSE20181 cohort: [RR], 71% in the high ICI group versus 50% in the low ICI group; chi-square test, *P <*0.05). Two immunotherapeutic cohorts confirmed that patients with the high ICI score had a higher responder rate to immunotherapy (GSE35640 cohort: [RR], 42.9% in the high ICI group versus 21.4% in the low ICI group; chi-square test, *P <*0.0001; GSE91061 cohort: [RR], 30.7% in the high ICI group versus 13.2% in the low ICI group; chi-square test, *P <*0.0001). In conclusion, in this study, we developed an ICI score to characterize the extensive immune cell infiltrating TME for BRCA, which could precisely predict the prognoses and response to chemotherapy and immunotherapy.

## Methods

### Data Accession

Discovery cohort: The RNA-seq data, clinical information, and somatic structural variation data of BRCA patients, were downloaded from The Cancer Genome Atlas (TCGA) (https://portal.gdc.cancer.gov) database. We enrolled 967 tumor samples and 112 adjacent normal samples with complete information including survival time, vital status, treatment strategies, RNA-seq data, and somatic variation data.

Validation cohort: We downloaded the RNA-seq data and clinical information of the Molecular Taxonomy of Breast Cancer International Consortium (METABRIC) cohort (14) from the cBio Cancer Genomics Portal website (www.cbioportal.org). A total of 1424 samples were enrolled to validation cohort with complete information including RNA-seq data, overall survival (OS), progression-free survival (PFS), and survival status.

Application cohorts: In total, we gathered 2 chemotherapeutic cohorts (GSE5462 and GSE20181) and 2 immunotherapeutic cohorts (GSE35640 and GSE91061). We downloaded the RNA-seq data and clinical data with these datasets from the Gene Expression Omnibus (GEO, https://www.ncbi.nlm.nih.gov/geo) database by the GEOquery R package (15). We removed these samples with absent or vague therapeutic responses.

### Evaluation of Immune Cell Abundances in the Discovery Cohort

To evaluate specific immune cell subsets, we systematically retrieved the issued studies and adopted an immune cell gene set that was proposed by Beibei Ru et al (16). This gene set was consisted of 742 gene signatures representing 28 immune cell subtypes including activated CD8 T cells, central memory CD8 T cells, effector memory CD8 T cells, activated CD4 T cells, central memory CD4 T cells, effector memory CD4 T cells, T follicular helper cells, gamma delta T cells, type 1 T helper cells, type 17 T helper cells, type 2 T helper cells, regulatory T cells, activated B cells, immature B cells, memory B cells, natural killer cells, CD56 bright natural killer cells, CD56 dim natural killer cells, myeloid derived suppressor cells, natural killer T cells, activated dendritic cells, plasmacytoid dendritic cells, immature dendritic cells, macrophages, eosinophils, mast cells, monocytes, and neutrophils (**Supplementary File 1**). The ssGSEA algorithm of the GSVA R package (17) was used to evaluate the relative immune cell abundances in each patients based on bulk RNA-seq data. The estimate R package (2) was used to asses immune and stromal cell contents for each sample. To comprehensively analyze the ICI status, we merged the immune cell abundance matrix and stromal cell content matrix for subsequent analyses. (**Supplementary File 2**).

### Comparison of the TME Between Normal and Tumor Tissues in the Discovery Cohort

To investigate TME differences between normal and tumor tissues, we compared the immune cell abundances and stromal cell contents of 112 normal tissues and their matched tumor tissues. We visualized the landscape of immune cell abundances and stromal cell contents through a heatmap by the pheatmap R package (18). The Wilcoxon test was used to compare the significant differences, and a two-tailed *P <*0.05 was considered to indicate a significant difference. Paired Student’s t test was used to compare the significant differences in PD1 and PD-L1 expression. The box plot drawn by the ggplot 2 R package (19) was used to show these data.

### Clustering for ICI Phenotypes With the Discovery Cohort

Based on the immune cell abundances and stromal cell contents of 967 tumor tissues, we performed unsupervised clustering by the consensusClusterPlus R package (20) to identify the ICI phenotypes. Unsupervised clustering depended on Euclidean distance and Ward’s linkage, and was repeated 1000 times to ensure the classification stability. The optimal number of clusters was determined by consensus matrix and relative change of area under a cumulative distribution function (CDF) curve. Finally, we obtained three distinct ICI phenotypes termed ICI Cluster ABC that divided the discovery cohort into three groups.

### Differentially Expressed Gene Analysis and Genotype Identification in the Discovery Cohort

To reveal the internal molecular characterizations with these three distinct ICI phenotypes, we performed pairwise comparisons to identify differentially expressed genes (DEGs) by the edgeR R package (21). Genes with a false discovery rate (FDR) adjusted *P value* < 0.05 and absolute value of |log_2_FC| (fold change) >1.3 were considered to statistically significant. To identify ICI genotypes, each Fragments Per Kilobase Million (FPKM) normalized DEGs expression value was standardized through log_2_ (expression value +1) formula across 967 BRCA samples. Additionally, the unsupervised clustering method proposed above was applied to identify ICI genotypes. A relative change in the area under the CDF curve and a consensus matrix were used to determine the optimal number of clusters. We obtained three ICI genotypes termed Gene Cluster ABC that classified the discovery cohort into three distinct groups.

### Gene Ontology Functional Annotations and ICI Score Development

The steps with ICI score development were processed as follows. First, we utilized a random forest algorithm to screen the most representative differentially expressed genes (MRDEGs) for these three distinct ICI genotypes by the randomForest R package (22). The random forest algorithm performed classifications and predictions to DEGs through multidecision trees. This algorithm ranked these DEGs by scoring their representativeness according to an accuracy or a Gini value. Here, the top one-third DEGs ranked by accuracy value were recognized as MRDEGs. Furthermore, GO functional annotations were used to investigate the functions of these MRDEGs by the clusterProfiler R package (23), and a *P* < 0.05 was considered to statistically significant. GO functional annotations included biological processes (BP), molecular functions (MF), and cellular components (CC). Next, we performed PCA using R software (version 4.0.2) to compute signature scores for each BRCA patient based on MRDEGs expression levels. Component 1 was extracted as signature scores according to a pervious study (13). Finally, we applied a method similar to gene expression grade index (24) to define the ICI scores for each patient as follows:


$$\text{ICI score }=\;\sum \text{PC}1i\;-\;\sum \text{PC}1_{j}$$

Where *i* is the signature score whose Cox efficiency is positive, and *j* is the signature score whose Cox efficiency is negative. Additionally, we calculated the ICI score of validation cohort based on the above steps using MRDEGs expression value. To investigate the prognostic value of the ICI score, we grouped the BRCA patients in the discovery cohort and validation cohort by an optimal cutoff area under the curve (AUC) of time-dependent receiver operating characteristic (ROC) analysis (25).

### Somatic Genetic Variation Data Analysis With the Discovery Cohort

The maftools R package (26) was used to count the total number of non-synonymous mutations to determine the TMB. To further investigate the prognostic value of TMB, we classified 967 tumor samples in the discovery cohort into high and low TMB subgroups based on an optimal cutoff AUC of ROC analysis. The maftools R package was used to visualize the landscape of the top 30 highest frequent alteration genes among the high and low ICI score subgroups. The chi-square test was used to detect the mutated genetic differences between high and low ICI score subgroups by the maftools R package, and a *P <*0.05 was regarded as statistically significance.

### Genomic and Clinical Dataset Analysis With Application Cohorts

To investigate the predictive role of the ICI score for therapeutic benefits, we filtered these MRDEGs expression value of the application cohorts. Next, we developed the ICI scores with application cohorts through the methodology proposed above. Finally, we grouped the patients who received corresponding therapies into high and low ICI score subgroups according to a median cutoff of the ICI score for further analysis.

### Statistical Analysis

All statistical analyses were conducted by R software (Version 4.0.2), and a *P* < 0.05 was considered to statistically significant. Univariate Cox regression analysis was performed to evaluate relationships between immune cells abundances and OS by survival R package (27). The comparisons of OS with specific groups were performed by the log-rank test of the survminer R package (28). The Kruskal–Wallis test and Wilcoxon test were utilized to examine the non-normalized distribution data. Student’s t test was used to compare normalized distribution data. The chi-square test was used to compare the categorical data.

## Results

### The TME Difference Between Tumor and Normal Tissues

**Figure 1:**
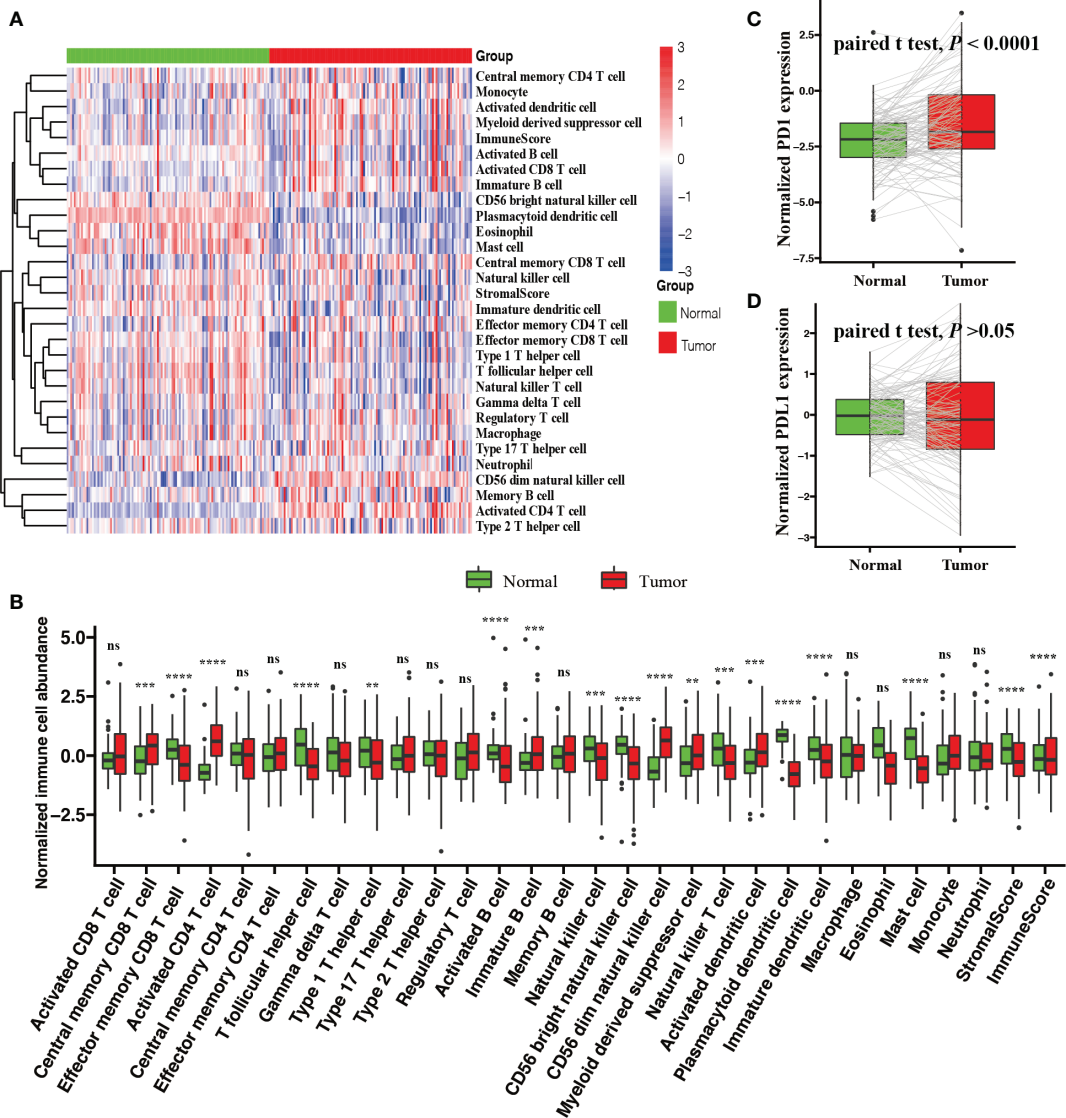
Comparison of the TME between tumor and normal tissues with BRCA. **(A)** Heatmap of the ICI landscape between tumor and normal tissues. **(B)** Box plot of normalized immune cell abundances (Kruskal–Wallis test, ns, no significance *P* > 0.05; **P < 0.01; ***P < 0.001; ****P < 0.0001). Comparison of the expression levels of two vital immune checkpoint molecules; **(C)** PD-1 and **(D)** PD-L1 (paired Student’s t test).

We compared the relative immune cell abundances and stromal cell contents of 112 normal tissues and their matched tumor tissues. Firstly, we visualized the relative immune cell abundances and stromal cell contents with tumor and normal tissues by a heatmap. Next, the Wilcoxon test was utilized to compare these immune cell abundances and stromal cell contents. We found that tumor tissues were remarkably characterized by high densities of central memory CD8 T cells, activated CD4 T cells, immature B cells, CD56 dim natural killer cells, myeloid-derived suppressor cells, and activated dendritic cells, but normal tissues were significantly characterized by high densities of effector memory CD8 T cells, T follicular helper cells, type 1 T helper cells, activated B cells, natural killer cells, CD56 bright natural killer cells, plasmacytoid dendritic cells, immature dendritic cells, mast cells, stromal score, and immune score (**Figures 1A, B**). In addition, we observed a significantly upregulated PD1 (paired Student’s t test; *P* < 0.0001, **Figure 1C**) and an undifferentiated PDL1 (paired Student’s t test; *P* > 0.05, **Figure 1D**) expression level in tumor tissues.

### Landscape of ICI Phenotypes With BRCA

**Figure 2:**
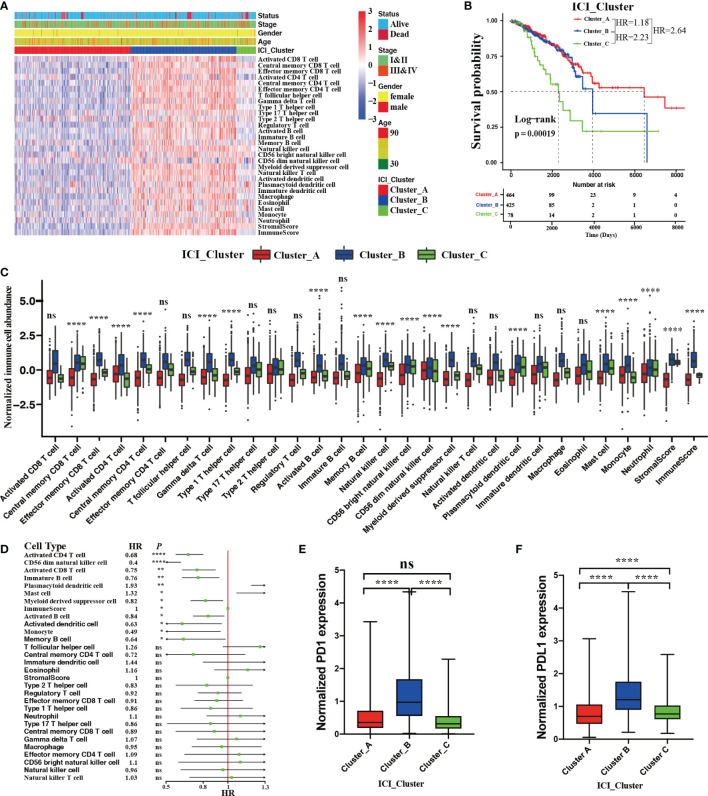
The ICI landscape in the TME of BRCA. **(A)** Unsupervised clustering for ICI phenotypes in the TME. The rows represent immune cells, and the columns represent BRCA samples. **(B)** The Kaplan–Meier curves showing OS (overall survival) outcomes in BRCA patients among these distinct ICI clusters. Log-rank test showing P = 0.00019. **(C)** The normalized immune cell abundances and stromal cell contents with distinct ICI clusters. (Kruskal–Wallis test, ns, no significance *P* > 0.05; ****P < 0.0001). **(D)** The prognostic role of immune cell abundance and stromal cell content. (Univariate Cox regression model, ns *P* > 0.05; *P < 0.05; **P < 0.01; ****P < 0.0001). Comparison of the expression level of two vital immune checkpoint molecules with these distinct ICI clusters; PD1 **(E)** and PD-L1 **(F)**. (Wilcoxon test, ns *P* > 0.05; ****P < 0.0001).

We performed an unsupervised clustering to systematically characterize the ICI patterns for BRCA using the relative immune cell abundances and stromal cell contents of tumor tissues. According to a consensus matrix and a relative change in area under the CDF curve, three divisions for TCGA-BRCA cohort was the best segregation that divided 967 BRCA patients into three heterogeneous phenotypes termed ICI Cluster A (464 patients), ICI Cluster B (425 patients), and ICI Cluster C (78 patients) (**Figure S1B–F**). **Figure 2A** sketched a landscape of BRCA clinical information and immune cellular distributions with these three ICI phenotypes. Prognostic analysis revealed a significant survival difference in these three ICI phenotypes (log-rank test; *P =* 0.00019, **Figure 2B**). Almost all immune cell types were remarkably different except for activated CD8 T cells, effector memory CD4 T cells, T follicular cells, type 17 T helper cells, type 2 T helper cells, regulatory T cells, immature B cells, natural killer T cells, activated dendritic cells, immature dendritic cells, macrophages, and eosinophils (Kruskal–Wallis test; *P* > 0.05, **Figure 2C**) in these ICI phenotypes. Among these distinct ICI phenotypes, ICI Cluster A patients, characterized by the lowest densities of almost all immune cell types except for activated CD4 T cells, CD56 dim natural killer cells, and monocytes, were associated with a favorable prognosis (median survival of 6472 days). The ICI Cluster B patients with a median survival time of 3842 days, were significantly marked by high densities of all immune cell types. Conversely, ICI Cluster C patients were witnessed the shortest OS (median survival of 2464 days) and were characterized by the lowest densities of activated CD4 T cells, CD56 dim natural killer cells, and monocytes. (**Figures 2B, C**).

We also performed univariate Cox regression analysis to reveal the prognostic roles of 28 immune cell abundances and stromal cell contents. As depicted in a forest plot, the abundances of activated CD4 T cells (hazard ratio [HR], 0.68; 95% confidence interval [CI], 0.58-0.8; *P* < 0.0001), CD56 dim natural killer cells (HR, 0.4; 95% CI, 0.26-0.62; *P* < 0.0001), activated CD8 T cells (HR, 0.75; 95% CI, 0.63-0.9; *P* < 0.01), immature B cells (HR, 0.76; 95% CI, 0.63-0.93; *P* < 0.01), myeloid derived suppressor cells (HR, 0.82; 95% CI, 0.7-0.96; *P* < 0.05), activated B cells (HR, 0.84; 95% CI, 0.72-0.97; *P*<0.05), activated dendritic cells (HR, 0.63; 95% CI, 0.42-0.95; *P* < 0.01), monocytes (HR, 0.49; 95% CI, 0.25-0.94; *P* < 0.05), and memory B cells (HR, 0.64; 95% CI, 0.42-0.98; *P* < 0.05) could serve as protective factors for BRCA patients. Conversely, the abundances of plasmacytoid dendritic cells (HR, 1.93; 95% CI, 1.19-3.13; *P* < 0.01) and mast cells (HR, 1.32; 95% CI, 1.07-1.62; *P* < 0.05) were the hazard factors for BRCA patients (**Figure 2D**). These findings were consistent with the worse OS with ICI Cluster C patients and the favorable OS with ICI Cluster A patients. Moreover, we compared the mRNA expression levels of two vital immune checkpoints (PD1 and PD-L1) in each ICI phenotypes by Wilcoxon test. We found that ICI Cluster A was remarkably characterized by the lowest PD1 (Wilcoxon test, *P* < 0.0001) and PD-L1 expression (Wilcoxon test*, P* < 0.0001). Conversely, ICI Cluster B was observed to have the highest PD1 (Wilcoxon test*, P* < 0.0001) and PD-L1 (Wilcoxon test*, P* < 0.0001) expression. Compared with ICI Cluster A, ICI Cluster C was observed to have the same PD1 (Wilcoxon test*, P* > 0.05) but significantly higher expression of PD-L1 (Wilcoxon test*, P* < 0.0001) (**Figure 2E, F**).

### Differentially Expressed Gene Analysis and Genotype Clustering

To investigate the intrinsic biological differences that led to distinct ICI phenotypes, we performed pairwise comparisons to identify the DEGs among these three ICI phenotypes. In total, 1183 DEGs were observed among these three ICI phenotypes (**Supplementary File 3**). As depicted in **Figure S2**, compared with ICI Cluster A, 265 upregulated and 94 downregulated mRNAs were identified in ICI Cluster B (**Figure S2A**). Compared with ICI Cluster B, 456 downregulated and 138 upregulated mRNAs were identified in ICI Cluster C (**Figure S2B**). Compared with ICI Cluster A, 49 upregulated and 605 downregulated mRNAs were observed in ICI Cluster C (**Figure S2C**). **Figure S2D** shows the gene relationships among these three ICI phenotypes. **Figure S2E** depicts a whole landscape of these 1183 DEGs expression levels. Next, an unsupervised clustering using these 1183 DEGs was performed to identify genotypes for BRCA patients. Three classifications dividing the discovery cohort into Gene Cluster A (437 patients), Gene Cluster B (342 patients), and Gene Cluster C (189 patients) were the best optimal according to the consensus matrix and relative change in the area under the CDF curve (**Figures S3A–E**).

**Figure 3:**
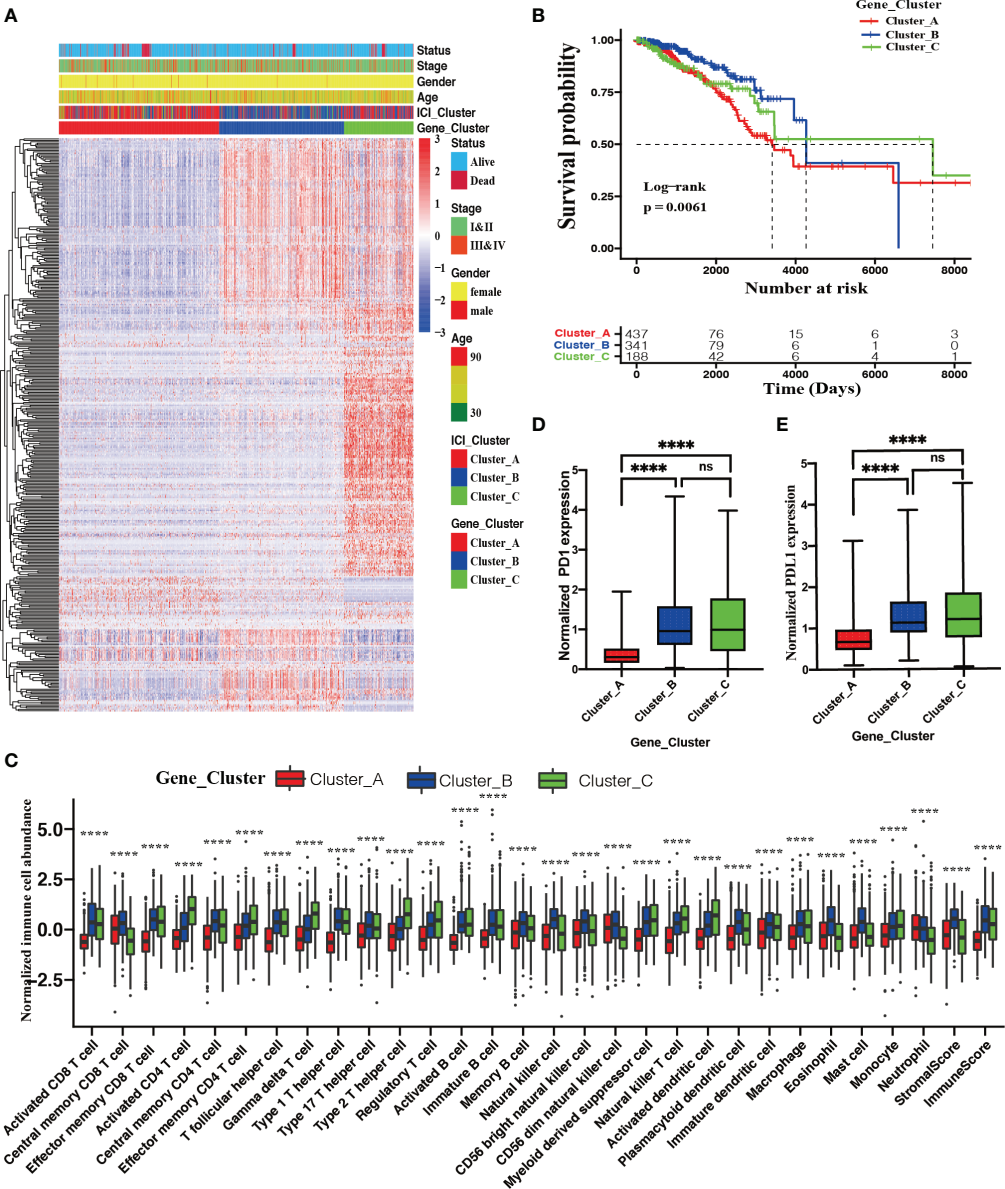
Identification of ICI genotypes. **(A)** Unsupervised clustering for DEGs among these distinct ICI clusters, which grouped BRCA patients into three Gene Clusters **(A–C)**. **(B)** The Kaplan–Meier curves showing OS outcomes in BRCA patients among these distinct gene clusters. Log-rank test showing P = 0.0061. **(C)** Normalized immune cell abundances and stromal cell contents in distinct gene clusters. (Kruskal–Wallis test, ****P < 0.0001). Comparison of the expression levels of two vital immune checkpoints **(D)** and PD-L1 **(E)** among these distinct ICI clusters; (Wilcoxon test, ns, no significance *P* > 0.05; ****P < 0.0001).

**Figure 3A** delineates a landscape of these 1183 DEGs and the clinical feature distributions with these 967 BRCA patients among distinct ICI genotypes. A significant difference in OS was observed among these three ICI genotypes (log-rank test; *P* = 0.0061, **Figure 3B**). Gene Cluster A patients characterized by the highest densities of neutrophils (Kruskal–Wallis test; *P* < 0.0001), suffered from the shortest median OS time with 3682 days. Gene Cluster B patients had a median OS time (4326 days), characterized by high densities of activated CD8 T cells, central memory CD8 T cells, effector memory CD8 T cells, central memory CD4 T cells, T follicular helper cells, type 1 T helper cells, immature B cells, memory B cells, natural killer cells, CD56 bright natural killer T cells, CD 56 dim natural killer T cells, plasmacytoid dendritic cells, immature dendritic cells, macrophages, eosinophils, mast cells, stromal score, and immune score. Conversely, Gene Cluster C patients enjoyed the longest median OS time (7345 days), characterized by high densities of activated CD4 T cells, effector memory CD4 T cells, gamma delta T cells, type 2 T helper cells, regulatory T cells, activated B cells, myeloid derived suppressor cells, natural killer T cells, activated dendritic cells, and monocytes (Kruskal–Wallis test; *P* < 0.0001, **Figures 3B, C**). Significantly upregulated PD1 and PD-L1 expression was observed in Gene Cluster B and Cluster C compared with Gene Cluster A (Kruskal–Wallis test; *P* < 0.0001, **Figures 3D, E**).

### Immune Cell Infiltration Score Development and Validation

First, we extracted 398 MRDEGs *via* a random forest algorithm (**Supplementary File 4**; **Figures S4A, B**). **Figure S4C** shows the whole landscape of these MRDEG expression levels with distinct ICI genotypes. To further validate the functions of these MRDEGs, we performed GO functional annotations. GO BP analysis showed that these MRDEGs were significantly enriched in the terms T-cell selection, lymphocyte costimulation, and positive regulation of T-cell proliferation. GO CC analysis showed that these MRDEGs were significantly enriched in the terms immunological synapse and alpha-beta T-cell receptor complex. GO MF analysis showed that these MRDEGs were significantly enriched in the terms T-cell receptor binding and cytokine receptor activity (**Figure S4D**, **Supplementary File 5**). Next, we utilized PCA to calculate the signature scores using these 398 MRDEG expression levels. Then, the patients were divided into two groups as high or low ICI scores basing on an optimal AUC cutoff value with ROC analysis. We also developed an ICI score and grouped the validation cohort using the methodology proposed above (**Supplementary File 6**).

**Figure 4:**
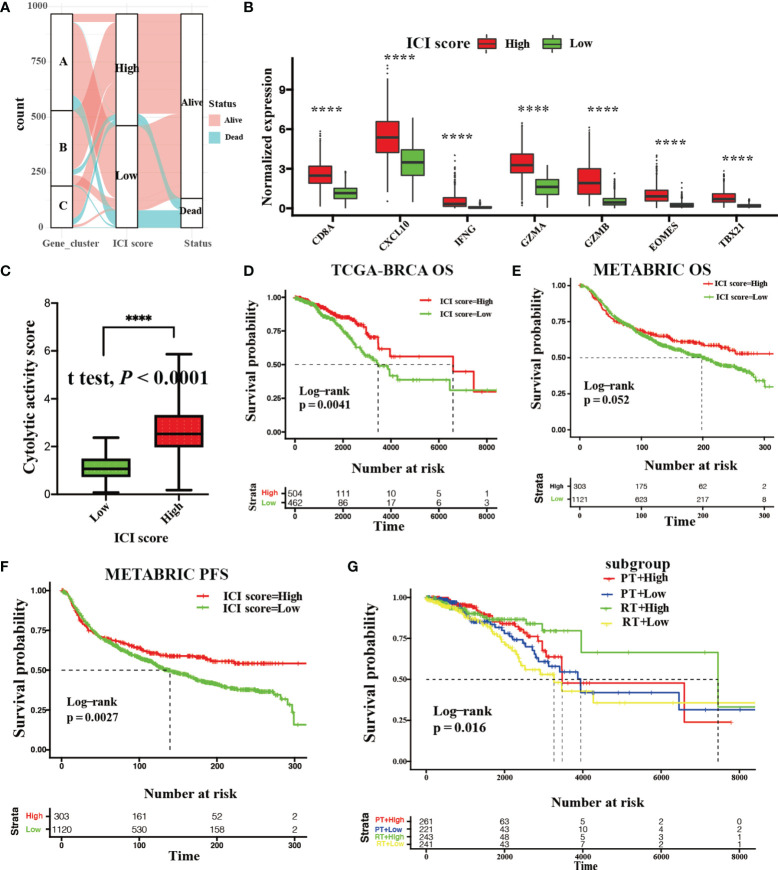
Development and validation with the ICI score. **(A)** Gene phenotype distribution of the ICI score and clinical outcomes. **(B)** Gene expression levels of effector T-cell infiltration (CD8A and CXCL10) and INF-γ-related cytotoxicity (IFNG, GZMA, GZMB, EOMES, and TBX21) between the high and low ICI score group. (Wilcoxon test ****P < 0.0001). **(C)** The cytolytic activity score between the high and low ICI score groups. (Student’s t test, ****P < 0.0001). **(D)** Kaplan–Meier curve showing OS outcomes between the high and low ICI score groups in the TCGA-BRCA cohort. Log-rank test showing *P* = 0.0041. **(E, F)** Kaplan–Meier curve showing OS outcomes (**E**; log-rank test, *P* = 0.052) and PFS outcomes (**F**; log-rank test, *P* = 0.0027.) with BRCA patients between the high and low ICI score groups in METABRIC cohort. **(G)** Kaplan–Meier curve showing OS outcomes of BRCA patients between the high and low ICI score groups treated with radiotherapy (RT) or chemotherapy (CT) in the TCGA-BRCA cohort (log-rank test, *P* = 0.016).

**Figure 4A** depicts the distribution of BRCA patients among these three genotypes. In addition, we assessed the level of effector T-cell infiltration (CD8A and CXCL10) and INF-γ-related cytotoxicity (IFNG, GZMA, GZMB, EOMES, and TBX21) for each ICI group based on a seven-gene panel designed in the POPLAR trial (29). Moreover, a cytolytic activity score calculated by the geometrical mean of PRF1 and GZMA mRNA expression levels was used to reflect the magnitude of the antitumor response (30). All of these eight parameters in the high ICI score group were remarkably higher than those in low ICI score group (Wilcoxon test; *P* < 0.0001, **Figure 4B**; Student’s t test; *P* < 0.0001, **Figure 4C**).

Kaplan–Meier survival with log-rank test was used to assess the relationships between OS and ICI score. We observed that BRCA patients with high ICI scores (median survival time: 6632 days) had significantly favorable OS versus patients with low ICI scores (median survival time: 3564 days) in the discovery cohort (log-rank test: *P* = 0.0041, **Figure 4D**). Additionally, a significant survival advantage of BRCA patients with high ICI scores was observed in PFS (log-rank test; *P* = 0.0027, **Figure 4F**) rather than OS (log-rank test; *P* = 0.052, **Figure 4E**) in the validation cohort. We also found that BRCA patients with high ICI scores who received radiotherapy or chemotherapy retained a significant survival advantage in discovery cohort (log-rank test; *P* = 0.016, **Figure 4G**).

### The Relationships Between the ICI Score and Somatic Genetic Variation

**Figure 5:**
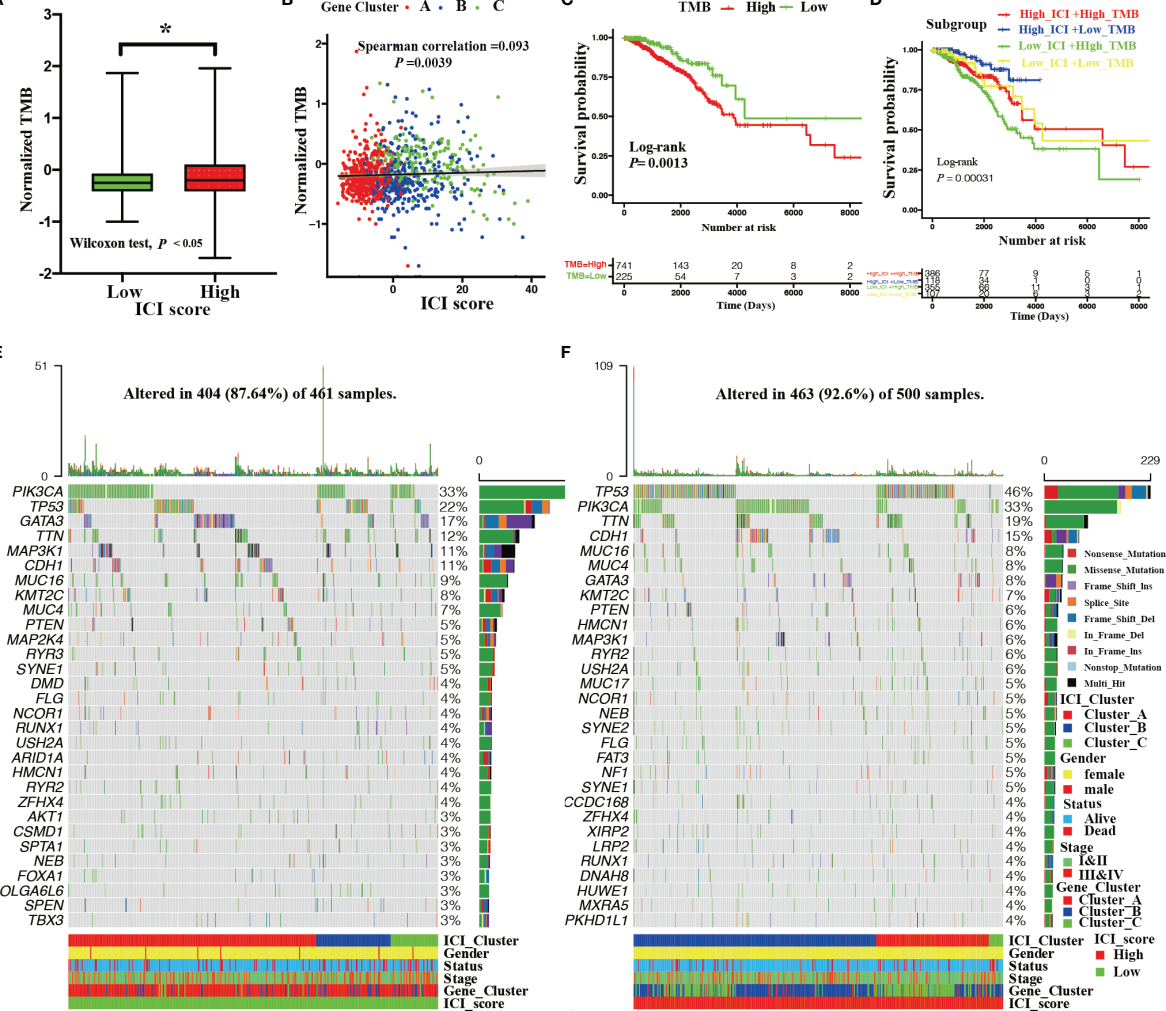
Correlation between ICI score and somatic mutation. **(A)** The TMB among the high and low ICI score groups (Wilcoxon test, *P < 0.05). **(B)** Correlation between the ICI score and TMB with distinct gene clusters (Spearman correlation test, *P* = 0.0039; spearman correlation coefficient = 0.093). **(C)** Kaplan–Meier curves showing OS outcomes of BRCA patients between high and low TMB group. Log-rank test showing *P* = 0.0013. **(D)** Kaplan–Meier curves for BRCA patients stratified by both TMB and ICI scores. Log-rank test showing *P* = 0.00031. **(E, F)** Landscape of the top 30 drive mutated genes for the low ICI score **(E)** and high ICI score groups **(F)**. Each column represents the individual patients and each row represents a drive mutated gene.

Previous studies have reported that tumor somatic genetic variation is related to cancer prognoses and determines therapeutic responses (31, 32). Inspired by these studies, we investigated the relationships between TMB and ICI score. First, a significantly elevated TMB was observed in BRCA patients with high ICI scores (Wilcoxon test; *P* < 0.05, **Figure 5A**). We also found that TMB was remarkably and positively correlated with ICI score (Spearman coefficient: R = 0.093, *P* = 0.0039, **Figure 5B**). To further study the prognostic value of TMB, we categorized BRCA patients into high TMB or low TMB subgroups based on an optimal cutoff of TMB value (0.37) with ROC analysis. As shown in **Figure 5C**, BRCA patients with low TMB had statistical survival advantage over those with high TMB (log-rank test; *P* = 0.0013). Additionally, hierarchical survival analysis revealed that this survival advantage was independent of TMB (log-rank test; *P* = 0.00031, **Figure 5D**).

The subsequent analyses assessed the distribution of driver somatic variant genes between the high and low ICI score subgroups. **Figure 5** shows the top 30 most frequently mutated genes with high (**Figure 5E**) and low (**Figure 5F**) ICI score groups. A total of 124 statistically different somatic variant genes were detected between high and low ICI score groups through the chi-square test conducted by the maftools R package (**Supplementary File 7**).

### The Role of the ICI Score in the Prediction of Therapeutic Benefits

**Figure 6:**
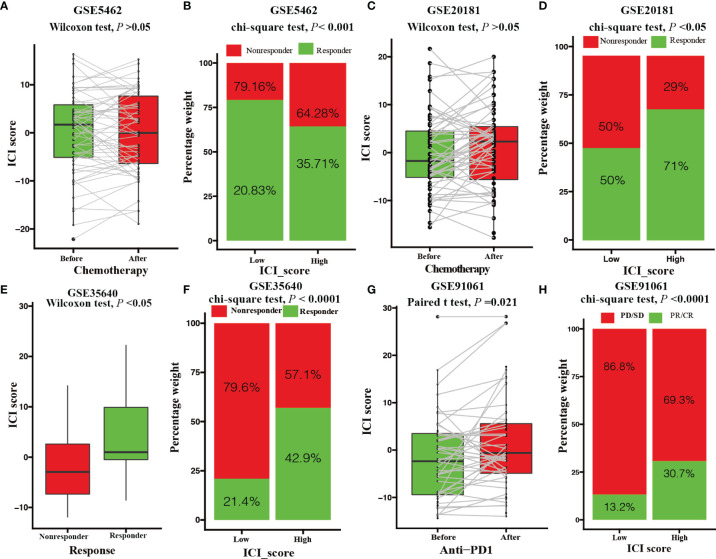
Value of the ICI score in predicting chemotherapeutic and immunotherapeutic benefits. **(A)** ICI score of patients with letrozole treatment before and after 10-14 days. (Paired Wilcoxon test; *P* > 0.05). **(B)** Rate of clinical response to letrozole among the low and high ICI score group after 10-14 days (chi-square test; *P* < 0.001). **(C)** ICI score of patients with letrozole treatment before and after 90 days. (paired Wilcoxon test; *P* > 0.05). **(D)** Rate of clinical response to letrozole in the low and high ICI score groups after 90 days (chi-square test; *P* < 0.05). **(E)** ICI score of melanoma patients with MAGE-A3 antigen treatment between responder and nonresponder groups (Wilcoxon test; *P* < 0.05). **(F)** Rate of clinical response to MAGE-A3 antigen treatment among the low and high ICI score groups (chi-square test; *P* < 0.0001). **(G)** ICI score of melanoma patients before and after PD-L1 antigen treatment (paired Student’s t test; *P* = 0.021). **(H)** Rate of clinical response to PD-L1 antigen among the low and high ICI score groups (chi-square test; *P* < 0.0001).

We assessed the role of ICI score in predicting chemotherapeutic benefits using the GSE5462 and GSE20181 datasets. The GSE5462 and GSE20181 datasets recorded microarray and clinical data from BRCA patients before and after letrozole treatment. As depicted in **Figure 6**, letrozole did not elevate the ICI score of BRCA patients (paired Wilcoxon test; *P* > 0.05, **Figures 6A, C**) but patients with high ICI scores had higher responder rate (GSE5462 cohort: responder rate [RR], 35.71% in the high ICI group versus 20.83% in the low ICI group; chi-square test, *P* < 0.001, **Figure 6B**; GSE20181 cohort: [RR], 71% in the high ICI group versus 50% in the low ICI group; chi-square test, *P* < 0.05, **Figure 6D**). We also investigated the immunotherapeutic benefit prediction role of the ICI score using GSE35640 and GSE91061 datasets. The GSE35640 and GSE91061 datasets recorded microarray and clinical data of melanoma patients with anti-MAGE-A3 and anti-PD1 treatments, respectively. We found that melanoma patients in the anti-MAGE-A3 responder group had higher ICI scores (GSE35640 cohort: paired Wilcoxon test; *P* < 0.05, **Figure 6E**). In contrast to chemotherapy, anti-PD1 treatment was found to elevate the ICI score of melanoma patients (GSE91061 cohort: paired Student’s t test; *P* < 0.0001, **Figure 6G**). Moreover, higher responder rates were observed in the high ICI score group with both anti-MAGE-A3 and anti-PD1 treatments (GSE35640 cohort: [RR], 42.9% in the high ICI group versus 21.4% in the low ICI group; chi-square test; *P* < 0.0001, **Figure 6F**; GSE91061 cohort: [RR], 30.7% in the high ICI group versus 13.2% in the low ICI group; chi-square test; *P* < 0.0001, **Figure 6H**).

## Discussion

An increasing number of studies have reported that the TME plays an indispensable role in tumor progression, prognosis, relapse, metastasis, and therapeutic response in BRCA. However, most studies have focused on a single TME component or regulator. A comprehensive TME characterization has not yet been recognized. Identifying distinct ICI patterns will promote our understanding of the TME and guide precise individual therapies.

In this study, based on the TCGA-BRCA cohort, we first investigated the difference in the TME between normal and tumor tissues. Our results revealed relatively sparse immune cells and an upregulated PD1 and an undifferentiated PDL1 expression level in BRCA tissues, which indicated that immune cell dysfunction and immune escape in the tumorous TME plays critical roles in tumorigenesis. The PD1/PDL1 pathway is regarded as a brake of immune system which attenuates the tumor-infiltrating lymphocytes (TILs) activation through increasing PDL1 expression on tumor cell surface (33). In line with our study, Uhercik et al. (34) also revealed an upregulated PD1 and an undifferentiated PDL1 expression level in BRCA tissues. However, Jun fang et al. (35) reported that mRNA expression level of PD1 was up-regulated but the PDL1 was down-regulated in BRCA tissues.

Our primary concerns were the TME with BRCA tissues, so we focused on an ICI with tumor tissues. Based on immune cell abundances and stromal cell contents with each tumor sample, we identified three ICI phenotypes for BRCA termed ICI cluster ABC. ICI Cluster A was characterized by sparse immune cells and stromal cell contents that corresponded to the immune-desert phenotype; ICI Cluster B was characterized by an activation of innate immunity and adaptive immunity that corresponded to the immune-inflamed phenotype; and ICI Cluster C was characterized by an activation of innate immunity and upregulated PD1 expression that corresponded to the immune-exclude phenotype. So far, few studies have reported the PD1 and PDL1 expression levels among those three ICI phenotypes in BRCA. But, in the Head and Neck Squamous Cell Carcinoma, Xinhai Zhang et al. (13) revealed a consistent PD1 and PDL1 expression levels with our study. In addition, we observed that the abundances of activated CD4 T cells, CD56 dim natural killer cells, activated CD8 T cells, immature B cells, myeloid-derived suppressor cells, monocytes, and memory B cells were associated with a better OS, but the abundances of plasmacytoid dendritic cells and mast cells were related with a worse OS, which was consistent with previous studies (30, 36–38). However, the prognostic analysis showed a mismatching OS order with these three ICI phenotypes. Although the immune-exclude phenotype was also characterized by high immune cell densities compared with immune-inflame phenotype, the abundant surrounding stromal cells protected these immune cells against penetrating the parenchyma, which suppressed their antitumor effects (39). Compared with the immune-desert phenotype, the OS disadvantage of the immune-inflame phenotype should be attributed to upregulated PD1 and PD-L1 expression, which inhibits immune cellular activation and increases the developmental T-cell exhaustion (40, 41). The mismatching OS orders with these three ICI phenotypes also implied that the ICI phenotypes cannot absolutely predict the prognosis. The extensive genetic alterations of these three ICI phenotypes might affect tumor prognosis.

These genetic alterations in tumor tissues changed the original patterns of intercellular interactions of infiltrating immune cells, which disturbed the balance of immunity tolerance and activity (42). Therefore, we hypothesized that ICI phenotype-associated genes could be novel biomarkers to determine suitable therapeutic strategies for BRCA patients, so we performed DEGs analysis and consensus clustering for genotypes. We found that Gene Clusters B and C had favorable OS with intact antigen presenting cells, CD8 T cells, and CD4 T cells indicating an immune-hot phenotype (43, 44). In contrast, the sparse immune and stromal cell distributions in Gene Cluster A imply an immune-cold phenotype. In addition, we observed that a survival advantage with Gene Cluster C might be associated with its high immune score and low density of neutrophils, eosinophils, and mast cells. In line with previous studies, we found that immune-hot phenotypic patients with a survival advantage had upregulated PD1 and PD-L1 expression, implying that immune-hot phenotypic BRCA patients might benefit more from immunotherapy (34, 45). In the clinical practice, the immune cell abundances of each sample can be detected by the Flow cytometry directly or the methodology proposed at present study. Basing on the immune cell abundances detected in the clinical practice and the characterizations of each ICI phenotype or genotype we identified, the doctors could group the patients in the matched ICI phenotype or genotype we proposed.

Through a random forest algorithm, we identified 394 MRDEGs for these ICI genotypes. As expected, a survival advantage of BRCA patients with high ICI score was observed in the discovery cohort and validation cohort. This survival advantage profited from its high level of effector T-cell infiltration, INF-γ-related cytotoxicity, and cytokine activity. Importantly, a hierarchical survival analysis of the discovery cohort revealed that the prognostic role of ICI score was independent of therapy strategies. Recent studies have explored a correlation between gene mutation and response or tolerance to therapies (46–49). We also investigated the correlation between the ICI score and variant frequency in multiple genes. Consistent with previous studies, therapy-sensitive somatic mutation genes were remarkably reduced in BRCA patients with low ICI scores (13). The correlation between the ICI score and TMB was 0.0093. A hierarchical analysis revealed that the prognostic value of the ICI score was independent of TMB. A low correlation coefficient and different predictive values implied that the ICI score and TMB were two distinct aspects of tumor biology.

Currently, chemotherapy remains a primary strategy for BRCA. Thus, it is necessary to assess the predictive value of the ICI score for chemotherapeutic benefits in BRCA patients. Our data indicated that chemotherapeutic agents could not elevate the ICI score, but patients with high ICI scores benefited more from chemotherapies. This phenomenon should be attributed to immunosuppression of chemotherapeutic agents. For a long time, immunotherapies have not been considered to a suitable strategy for BRCA due to low immunogenic peptide presentation (50). The phase III Impassion 130 trial reported that immune checkpoint block gained promising clinical efficacy in extending survival time with BRCA patients, which news recaptured researchers’ attention (51). Despite a successful application of immunotherapies across a broad range of cancers, only a few patients could benefit from it. Even the issued guidelines with Society for Immunotherapy of Cancer had emphasized that a population suitable for immunotherapies should be further identified (52). Here, our data revealed that patients with high ICI scores benefited more from immunotherapies, which indicated that ICI scores could guide immunotherapies. Generally, the ICI score we developed could be a robust tool to guide individual treatments for cancer.

Our study also has several limitations. First, a PFS not OS confirmed the prognostic value of the ICI score in the validation cohort. Second, due to the absent of public BRCA immunotherapeutic cohorts, we validated the prognostic value with ICI score for immunotherapies using two melanoma cohorts. Third, a comprehensive study integrating ICI patterns, clinical information, and somatic mutation information was solely performed in the TCGA-BRCA cohort. However, the accessibility of these data to BRCA patients is insufficient in the validation cohort, thus, we failed to validate all of our findings in multiomics. Considering that these 967 TCGA-BRCA tumor samples could sufficiently contain all ICI patterns in the TME, we did not merge the RNA-seq datasets from different sequencing platforms to avoid batch effects. Last, we failed to perform RNA sequencing with an internal validation cohort for research funding limitations. Therefore, we included 1424 samples with complete information in the validation cohort to overcome this shortcoming. Further comprehensive studies integrating multiomics data are still prospective in this field.

In conclusion, by applying bioinformatics and multiomics analyses, we identified there ICI patterns and developed an ICI score to characterize the extensive immune cell-infiltrating TME for BRCA, which could precisely predict the clinical outcome and response to chemotherapy and immunotherapy.

## Data Availability Statement

The datasets presented in this study can be found in online repositories. The names of the repository/repositories and accession number(s) can be found in the article/**Supplementary Material**.

## Ethics Statement

Ethical review and approval was not required for the study of human participants in accordance with the local legislation and institutional requirements. Written informed consent from the participants was not required to participate in this study in accordance with the national legislation and the institutional requirements.

## Author Contributions

LL designed this study, performed the statistical analysis, and prepared the manuscript; The author approved the final version for submission.

## Conflict of Interest

The author declares that the research was conducted in the absence of any commercial or financial relationships that could be construed as a potential conflict of interest.

## Publisher’s Note

All claims expressed in this article are solely those of the authors and do not necessarily represent those of their affiliated organizations, or those of the publisher, the editors and the reviewers. Any product that may be evaluated in this article, or claim that may be made by its manufacturer, is not guaranteed or endorsed by the publisher.

## Supplementary Material

Supplementary Figure 1 The workflow of study design and clustering for ICI phenotypes. The workflow diagram of study design **(A)**. Consensus matrix with 2 **(B)**, 3 **(C)**, 4 **(D)**, and 5 **(E)** divisions for BRCA samples. **(F)** Scree plot of relative change in area under cumulative distribution function (CDF) curve.

Supplementary Figure 2 Differentially expressed genes (DEGs) identification with these distinct ICI phenotypes. The volcano plot of DEGs with ICI Cluster A versus Cluster B **(A)**, ICI Cluster B versus Cluster C **(B)**, and ICI Cluster A versus Cluster C **(C)**. The red dots represent upregulated genes, while the green dots represent downregulated genes. **(D)** The relationships between the DEGs among distinct ICI phenotypes. **(E)** The expressional landscape with DEGs.

Supplementary Figure 3 Clustering for ICI genotypes. Consensus matrix with 2 **(A)**, 3 **(B)**, 4 **(C)**, 5 **(D)**, and 6 **(E)** divisions for the TCGA-BRCA cohorts. **(E)** Scree plot with relative change in area under cumulative distribution function (CDF) curves. The elbow indicates that three divisions for the TCGA-BRCA cohorts were the best optimal.

Supplementary Figure 4 Screening of MRDEGs. **(A)** The relationships between the predictive error rate and the number of trees with random forest. The three lines in different colors represent three distinct ICI genotypes. **(B)** The top 30 MRDEGs according to mean decreasing accuracy and decreasing Gini. **(C)** The landscape of these top 398 MRDEGs. The rows represent MRDEGs and the columns represent BRCA samples. **(D)** The bubble plot of GO functional annotations with MRDEGs.

Supplementary File 1 The immune cells gene set.

Supplementary File 2 Relative Fractions of tumor-infiltrating immune cells of 112 normal tissues and 968 tumor tissues with TCGA-BRCA patients.

Supplementary File 3 Different expressional genes of these distinct ICI phenotypes.

Supplementary File 4 The representativeness of DEGs with descending order by random forest algorithm and top 398 most representative DEGs expression level.

Supplementary File 5 GO functional annotations with these top 398 most representative DEGs.

Supplementary File 6 ICI score of TCGA-BRCA cohort, METABRIC cohort, immunotherapeutic cohorts, and chemotherapeutic cohorts.

Supplementary File 7 The somatic gene mutations between high and low ICI groups with TCGA-BRCA cohort.

## Abbreviations

BRCA, Breast Cancer; TME, Tumor microenvironment; ICI, Immune cells infiltration; ssGSEA, single sample gene set enrichment analysis; OS, overall survival; PFS, free progress survival; PCA, principal component analysis; CDF, cumulative distribution function; TMB, tumor mutation burden; DEGs, differential expressed genes; MRDEGs, the most representative differential expressed genes; TCGA, The Cancer Genome Atlas; GEO, Gene Expression Omnibus; AUC, Area under curve; ROC, receiver operating characteristic; PD1, Programmed cell death-1; PD-L1, Programmed cell death-ligand-1; MAGE-A3, Melanoma-associated antigen 3; METABRIC, Molecular Taxonomy of Breast Cancer International Consortium; RR, Responder rate; HR, Hazard ratio; CI, Confidence interval; GO, Gene Ontology; CC, Cellular components; BP, Biological processes; MF, Molecular functions; TILs, Tumor-infiltrating lymphocytes.
