# Supplementary figures and images for: Tumor Microenvironment Characterization in Breast Cancer and an Immune Cell Infiltration Score Development, Validation, and Application
Source: Front Oncol. 2022 Jun 27;12:844082. doi: 10.3389/fonc.2022.844082 (PMC9273207; doi:10.3389/fonc.2022.844082)

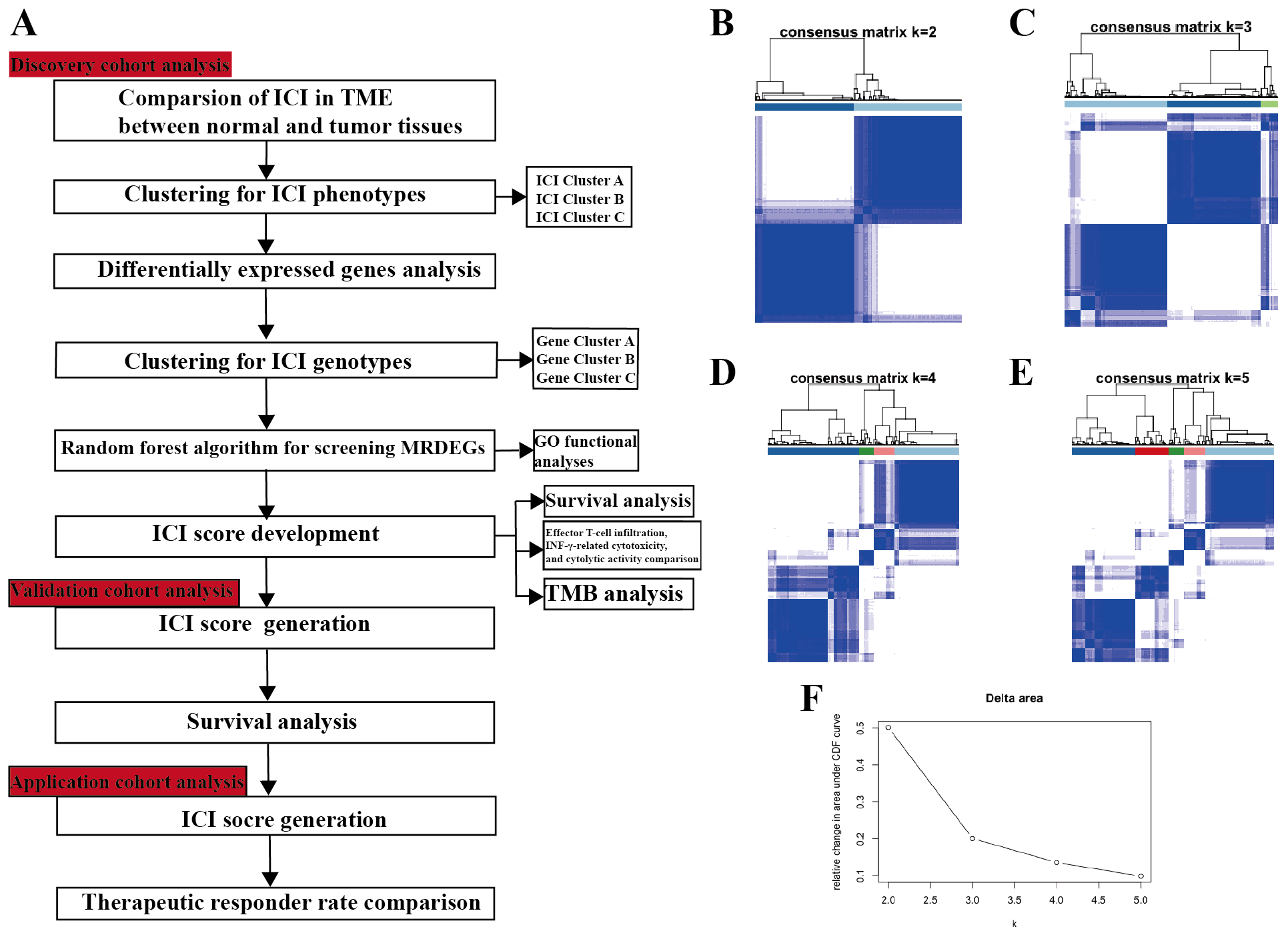

Supplement: Supplementary Figure 1 — The workflow of study design and clustering for ICI phenotypes. The workflow diagram of study design (A). Consensus matrix with 2 (B), 3 (C), 4 (D), and 5 (E) divisions for BRCA samples. (F) Scree plot of relative change in area under cumulative distribution function (CDF) curve. [file Image_1.tif]

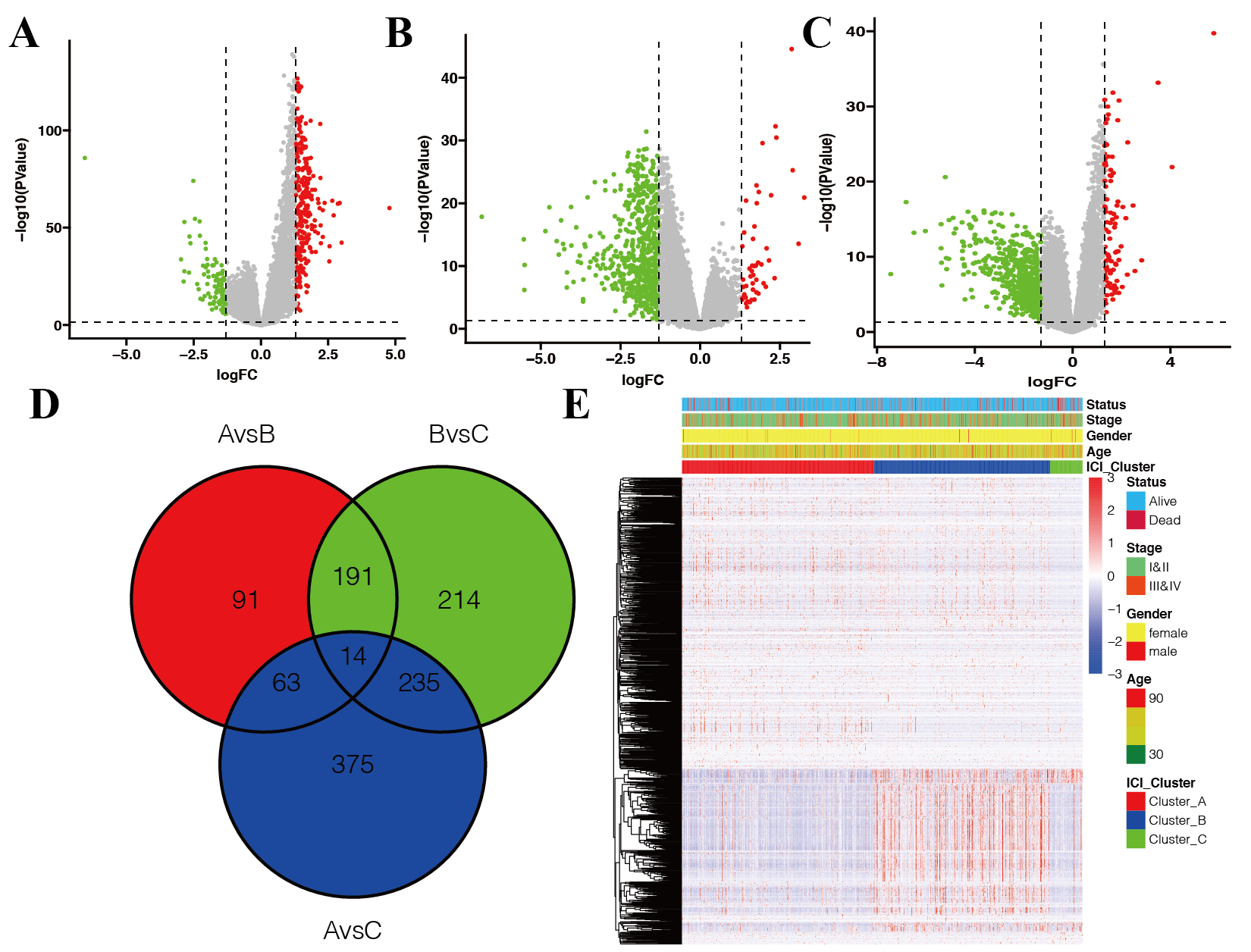

Supplement: Supplementary Figure 2 — Differentially expressed genes (DEGs) identification with these distinct ICI phenotypes. The volcano plot of DEGs with ICI Cluster A versus Cluster B (A), ICI Cluster B versus Cluster C (B), and ICI Cluster A versus Cluster C (C). The red dots represent upregulated genes, while the green dots represent downregulated genes. (D) The relationships between the DEGs among distinct ICI phenotypes. (E) The expressional landscape with DEGs. [file Image_2.tif]

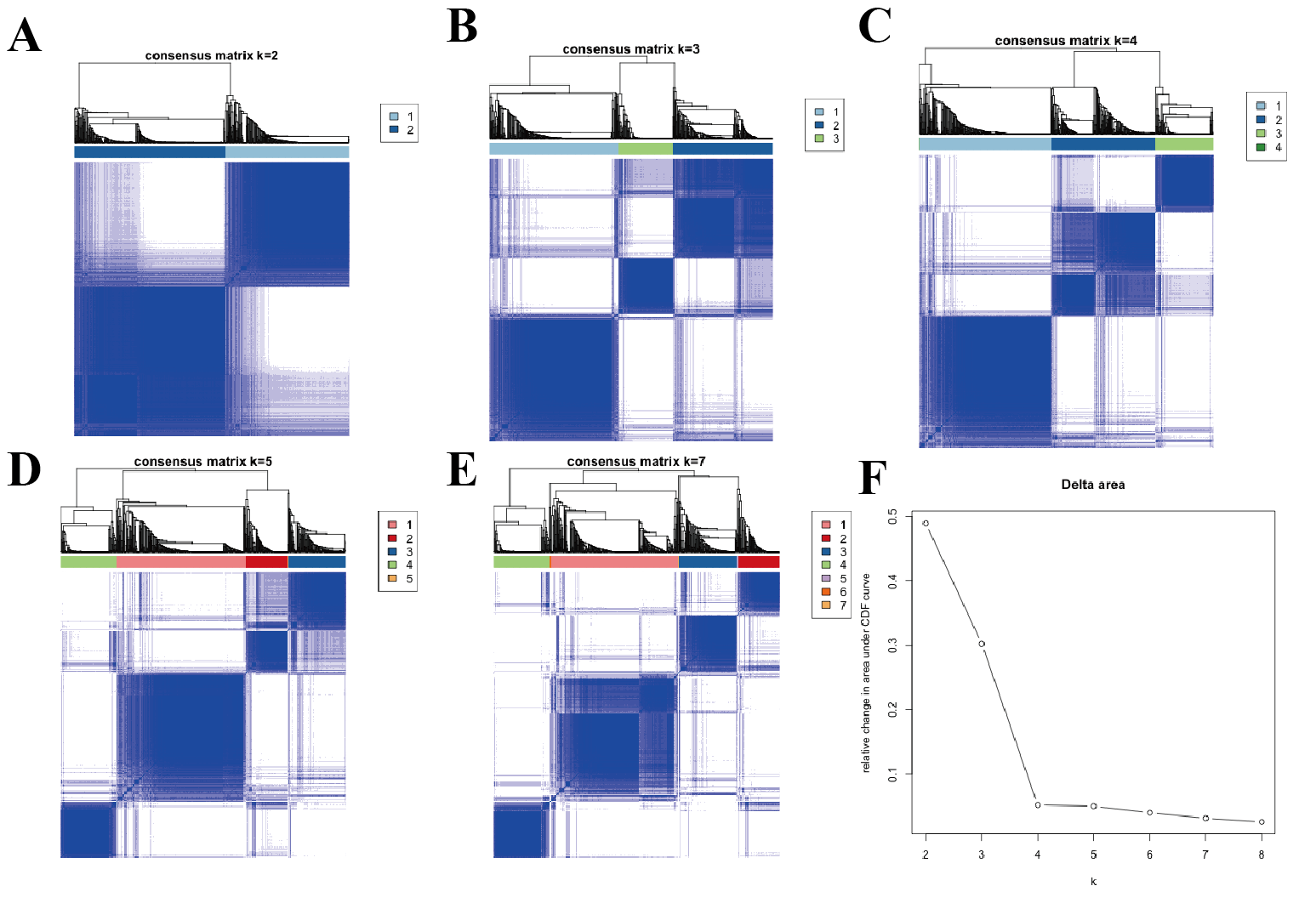

Supplement: Supplementary Figure 3 — Clustering for ICI genotypes. Consensus matrix with 2 (A), 3 (B), 4 (C), 5 (D), and 6 (E) divisions for the TCGA-BRCA cohorts. (E) Scree plot with relative change in area under cumulative distribution function (CDF) curves. The elbow indicates that three divisions for the TCGA-BRCA cohorts were the best optimal. [file Image_3.tif]

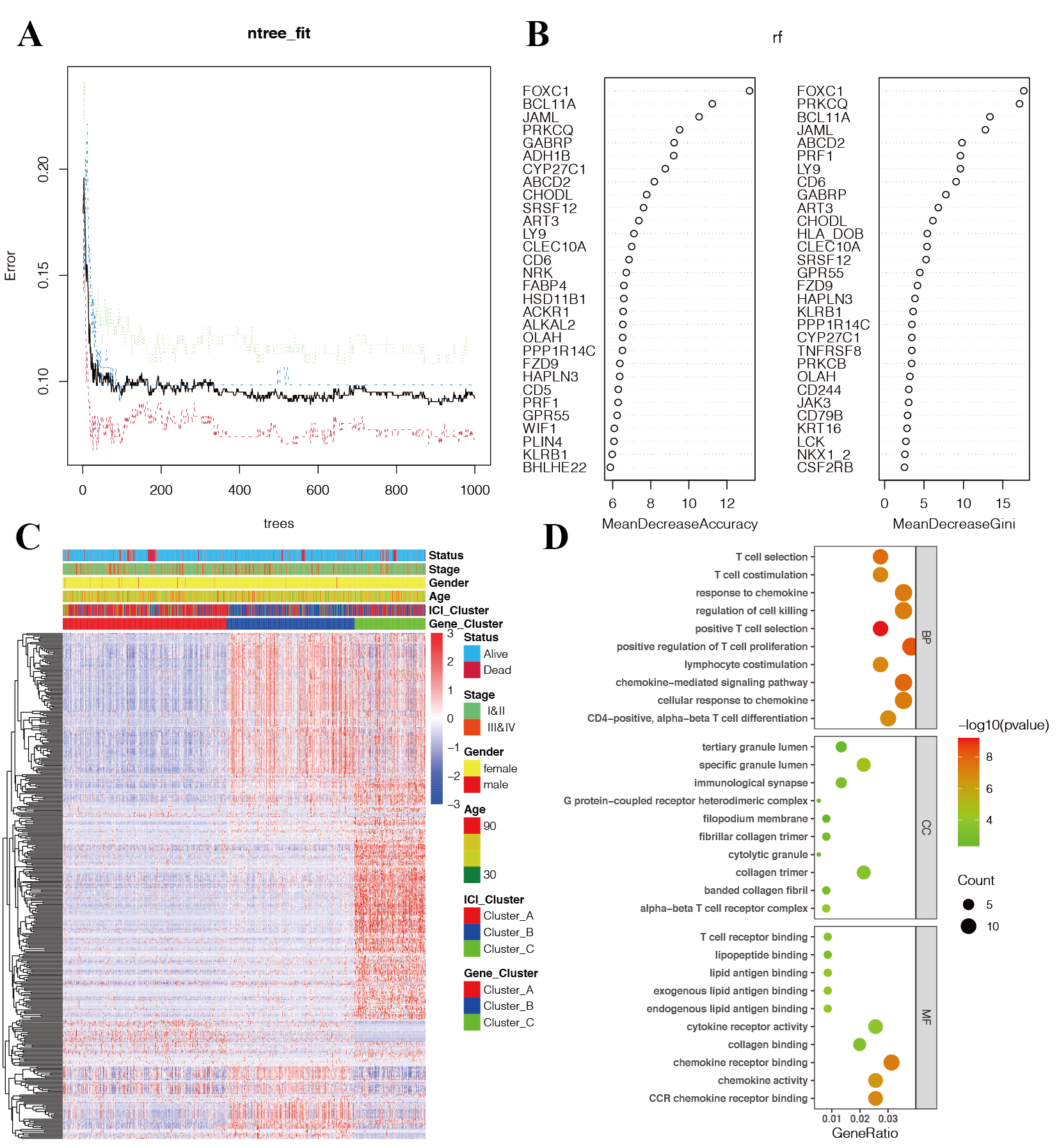

Supplement: Supplementary Figure 4 — Screening of MRDEGs. (A) The relationships between the predictive error rate and the number of trees with random forest. The three lines in different colors represent three distinct ICI genotypes. (B) The top 30 MRDEGs according to mean decreasing accuracy and decreasing Gini. (C) The landscape of these top 398 MRDEGs. The rows represent MRDEGs and the columns represent BRCA samples. (D) The bubble plot of GO functional annotations with MRDEGs. [file Image_4.tif]
